# Editorial: Diagnosis and treatment of non-functioning pituitary tumors

**DOI:** 10.3389/fendo.2025.1558988

**Published:** 2025-04-03

**Authors:** Aydin Sav

**Affiliations:** Yeditepe University, School of Medicine, Department of Medical Pathology, Istanbul, Türkiye

**Keywords:** editorial, frontiers endocrinology, pituitary endocrinology, diagnosis, treatment, nonfunctioning pituitary adenoma (NFPA), molecules

As defined in the 2017 WHO classification, pituitary adenoma evolved into PitNET in the more recent 2022 WHO classification. In the relevant literature, PA (pituitary adenoma) and PitNET (pituitary neuroendocrine tumor) have been used interchangeably, and NFPA and NF-PitNET for non-functioning pituitary adenoma. Non-functioning pituitary adenoma (NF PitNET) is a relatively rare anterior pituitary tumor originating from hormone-producing neuroendocrine cells of the adenohypophysis. Clinical implications depend on the mass effect of sellar or parasellar locations unaccompanied by any clinical signs of hormonal hypersecretion. The most typical manifestations are visual disturbances, headache, cranial nerve dysfunction, and hypopituitarism. They might be discovered incidentally (1). Non-functioning pituitary adenomas (NFPAs) are among the most common tumors in the sellar region. They account for 15-30% of pituitary adenomas. These lesions do not cause hyperpituitarism. In the majority of cases, they are found incidentally (particularly microadenomas), or their mass effect gives rise to compressive symptoms such as headache and visual field defects, along with hypopituitarism. The clinical term “non-functioning” is not a diagnosis but a description of a clinical scenario with many differential diagnoses. The most common lesion is a gonadotroph tumor, as described above; they constitute approximately 70–75% of clinically non-functioning pituitary adenomas (1, 2). Non-functioning pituitary adenomas are segregated depending on age. Silent PitNETs are silent corticotroph tumors among adult tumors (1). Immature PIT1-lineage tumors are predominantly seen in younger age groups among non-functioning NFPAs (3). Clinically silent corticotrophs and silent PIT1-lineage tumors, such as non-functioning immature PIT1-lineage tumors, show a biologically aggressive pattern (4). Diagnosis is usually made in the context of mass effect due to a macroadenoma or, increasingly, fortuitously during imaging performed for unrelated purposes; the latter case is known as pituitary incidentaloma. Surgery is indisputably indicated in the case of tumor syndrome. However, other aspects of NFPA (hormonal work-up, follow-up, especially postoperative follow-up, management of remnant or recurrence, the special case of incidentaloma, or apoplexy) remain controversial (5). Invasive non-functional pituitary adenomas account for 35% of NFPAs. Although a vast number of ongoing studies have investigated the complete underlying molecular mechanisms of the invasive potential of NFPA, they have not yet deciphered the underlying molecular mechanisms of invasiveness (6). Diagnostic NF-PitNET issues are problematic in pathology and clinical science. Chang et al. investigated bioactive molecules carrying small vesicles and exosomes, imparting RNA to regulate recipient cells. Exosomes bear a specific microRNA, hsa-miR-1180, as an early tumor NF-PitNET biomarker. The hsa-miR-21-5p accelerates distant osteogenesis in GHPA. Exosomal protein transcripts are potential invasive biomarkers, such as MMP1, N-cadherin, CDK6, RHOU, INSM1, and RASSF10. In review, Tumor suppressors in the exosomes represent novel therapeutic applications of exosomes, including long non-coding RNA (lncRNA) H19, miR-149-5p, miR-99a-3p, and miR-423-5p. Divergent contents of exosomes, such as long noncoding RNA (lncRNA) H19, miR-149-5p, miR-99a-3p, and miR-423-5p, participate in cells of NF-PitNET mechanisms are planned to be used in clinical diagnosis and their treatment.

A recent WHO classification emphasizes the importance of transcription factors (TFs) in the diagnosis and treatment of neoplasms originating from the pituitary gland, namely PitNETs. TFs are referred to as their molecular profiles, steroidogenic factor (SF-1), T-box family member TBX19 (TPIT), and POU class 1 homeobox 1 (Pit-1). Woo et al. investigated a selected cohort of NF-PitNET cases (n=113) and classified the profiles based on TF distribution. The distribution of NF-PitNET TF profiles showed that the majority of NF-PitNETs were SF-1-lineage tumors (58.4%), followed by TPIT-lineage tumors (18.6%), tumors with no distinct lineage (16.8%) and Pit-1-lineage tumors (6.2%). COX regression showed no lineage difference between SF1 and PitNET without any evidence of lineage profile. There was no correlation between tumor volume and PitNET without any lineage, and they were accepted as independent predictors of a composite of residual or recurrent disease. The 2022 WHO classification adds to the importance of TFs and lineage-based systems for subtyping in the prediction and prognosis of NF-PitNETs.

As previous studies have shown, NF-PitNETs have little response to therapeutics. The study by Gil et al. linked epithelial-mesenchymal transition (EMT) to resistance to medical treatment in a group of pituitary adenomas. Their research revealed the potential usefulness of medical treatment for NF-PitNETs by closely examining the expression of somatostatin receptors and dopamine-associated genes. Moreover, SNAI1, SNAI2, Vimentin, KLK10, PEBP1, Ki-67 and SSTR2 were associated with invasive NF-PitNET. Genetically, PEBP1 overexpression was remarkably high in recurrent NF-PitNETs, giving the impression that it could predict growth recurrence with 100% sensitivity but only 43% specificity. The EMT phenomenon was more common in NF-PitNETs than in GH-secreting pituitary tumors.

In conclusion, EMT has a place in NF PitNETs. SSTR3 targeting could be a potential therapeutic target in selected cases other than corticotropinoma with low expression of SSTR3. Due to its presence, this molecule could be a predictive indicator of recurrence in NF-PitNETs.

The well-defined preoperative and postoperative complications of pituitary adenoma patients were analyzed based on their various signs, such as headache, vomiting, and visual field defects based on improvement. Wang et al. looked at the difference between young (<73 years) and older (>73 years) patients classified as having functioning and non-functioning pituitary adenomas. The majority of non-functioning pituitary adenoma patients were elderly patients (73,7%) who had a high degree of suprasellar invasion but a low degree of parasellar invasion (P<0.0001). Older patients suffered from a high incidence of suprasellar invasion, poor postoperative visual improvement, a higher rate of intratumoral bleeding, and comorbid postoperative complications, such as cerebrospinal fluid leakage and fever. In conclusion, specific signs and symptoms associated with the postoperative period of the elderly NFPA cohort needed instant surgical attempts to improve their health status.

NF-PitNETs are resistant to medical therapeutics. Therefore, the intention is to provide knowledge of the biological properties of PitNETs. Wu et al. explored immune infiltration-associated differentially expressed genes (DEGs) by deciphering high/low immune scores calculated by the ESTIMATE algorithm. Another technic, WGCNA, analyze to construct a coexpression network of immune cell-related genes. Random forest analysis selection analyzes candidate genes associated with invasion. Finally, external validation verified gene expressivity using quantitative real-time polymerase chain reaction (qRT–PCR). In conclusion, the 8-gene (BMP6, CIB2, FABP5, HOMER2, MAML3, NIN, PRKG2, and SIDT2) classification model was correlated with acceptable values verified by qRT–PCR. All these pivotal roles in invasion and progression can be used as major targets for immunotherapy.
